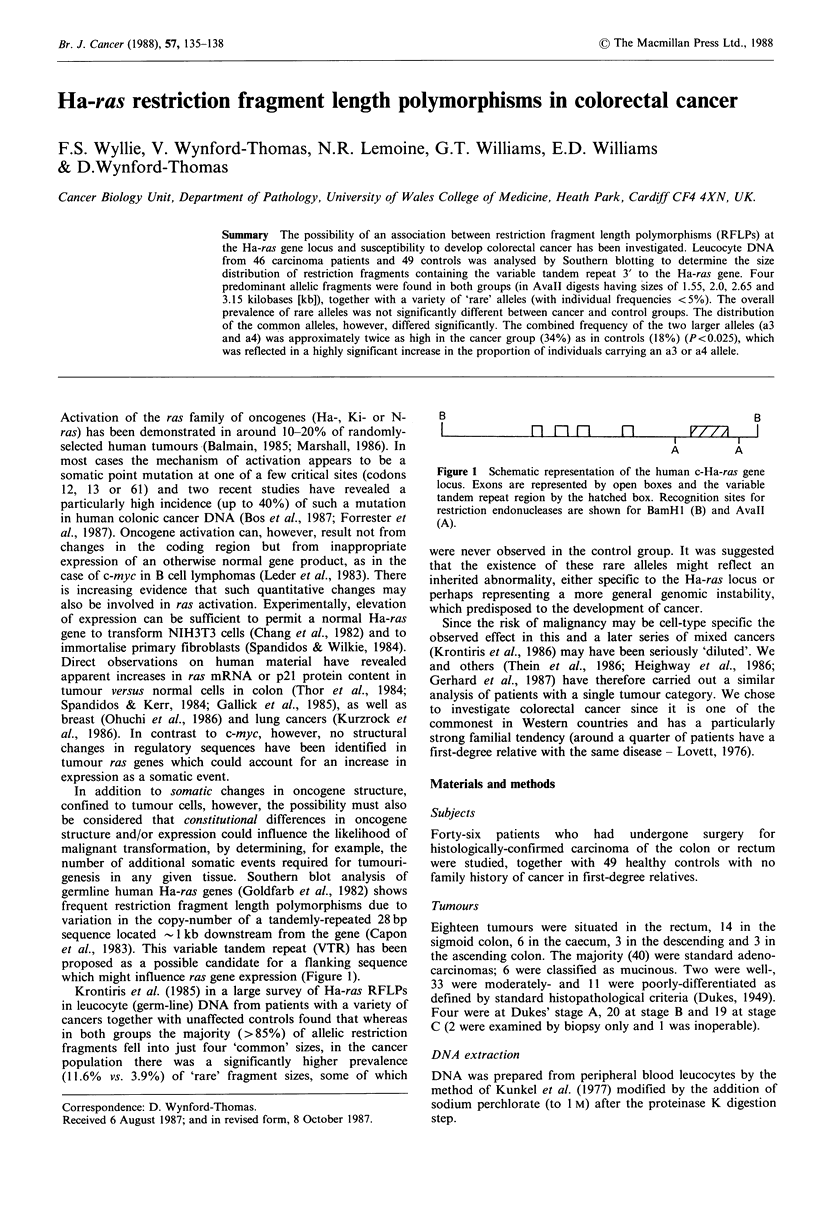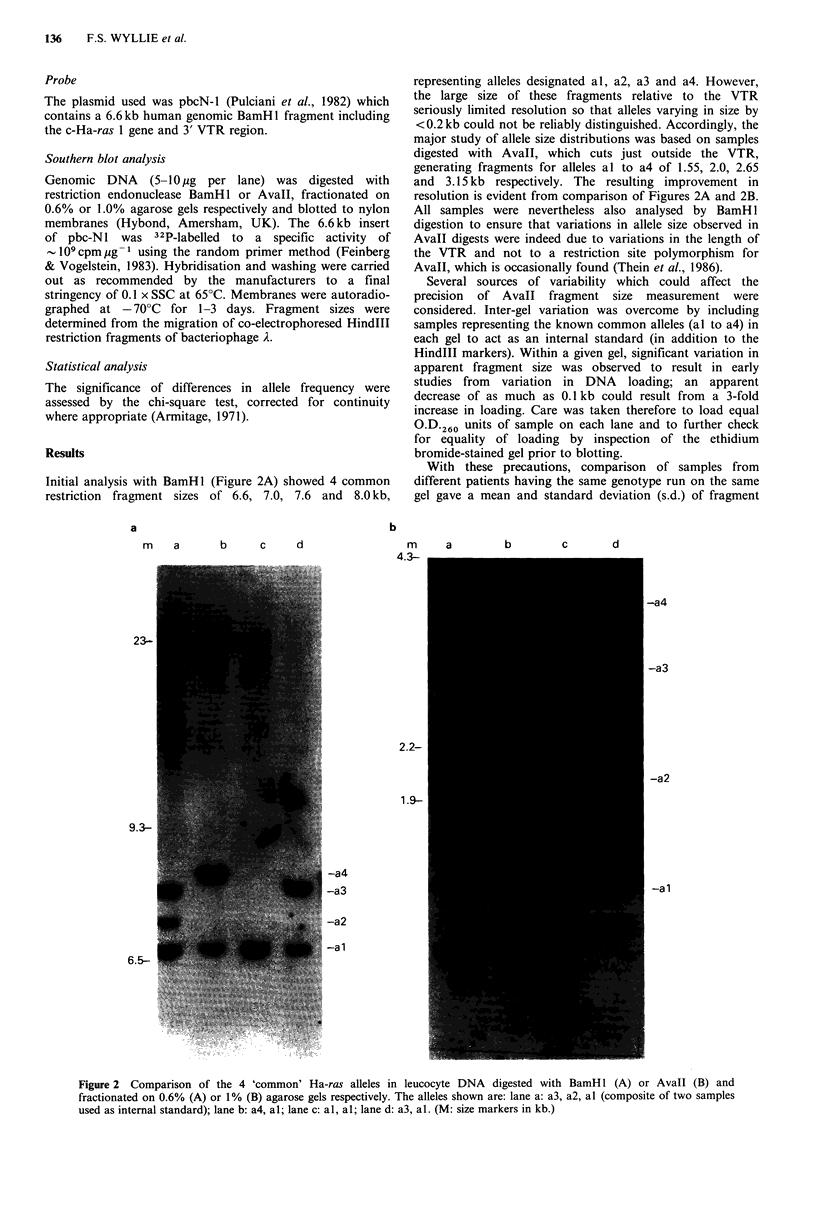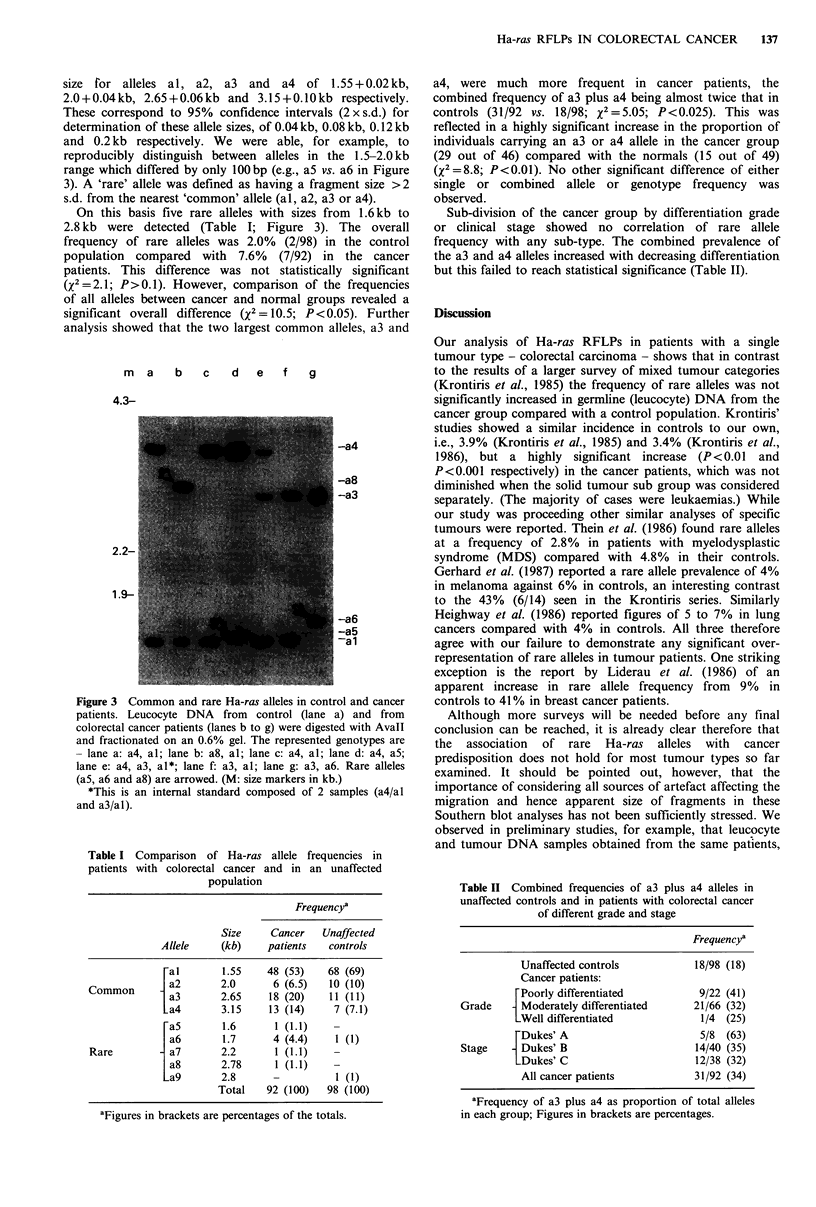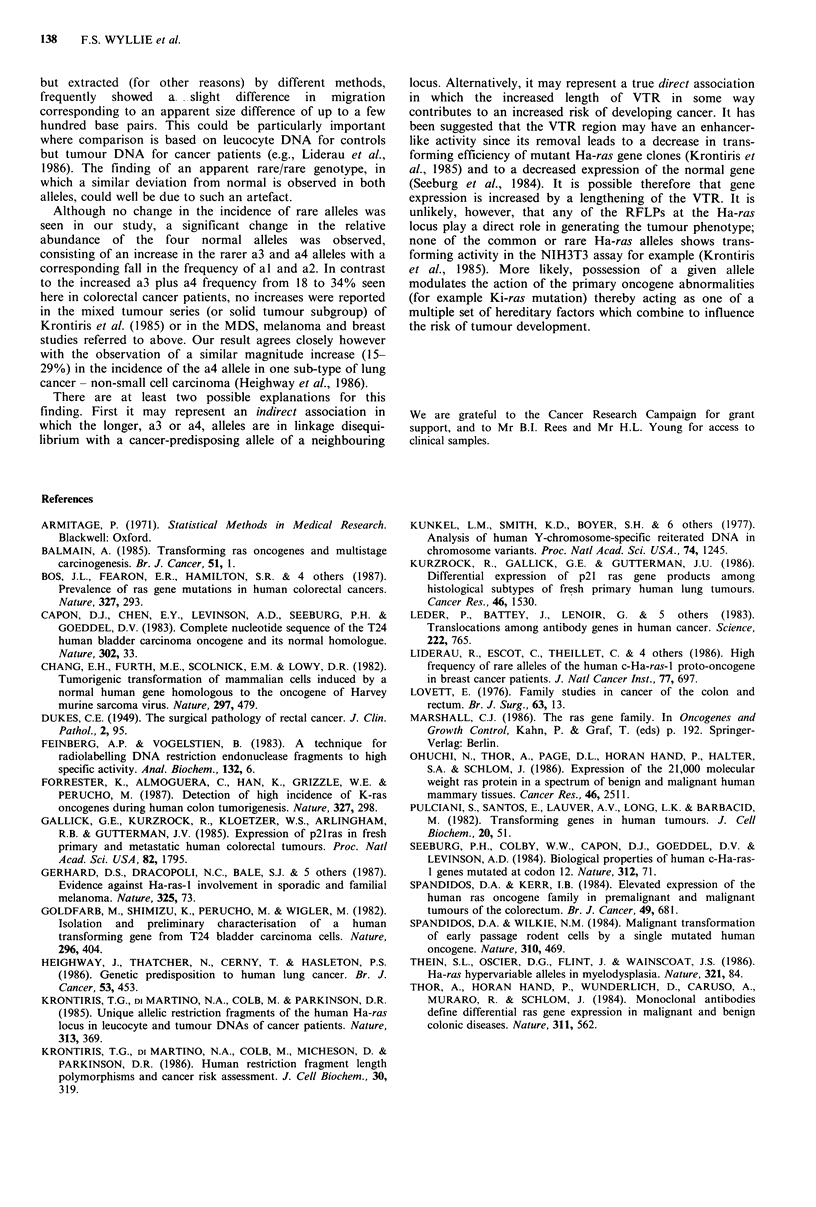# Ha-ras restriction fragment length polymorphisms in colorectal cancer.

**DOI:** 10.1038/bjc.1988.28

**Published:** 1988-02

**Authors:** F. S. Wyllie, V. Wynford-Thomas, N. R. Lemoine, G. T. Williams, E. D. Williams, D. Wynford-Thomas

**Affiliations:** Department of Pathology, University of Wales College of Medicine, Heath Park, Cardiff, UK.

## Abstract

**Images:**


*Br. J. Cancer* (1988), **57**, 135–138 © The Macmillan Press Ltd., 1988

### Ha-*ras* restriction fragment length polymorphisms in colorectal cancer

F.S. Wyllie, V. Wynford-Thomas, N.R. Lemoine, G.T. Williams, E.D. Williams & D.Wynford-Thomas

*Cancer Biology Unit, Department of Pathology, University of Wales College of Medicine, Heath Park, Cardiff CF4 4XN, UK.*

**Summary** The possibility of an association between restriction fragment length polymorphisms (RFLPs) at the Ha-*ras* gene locus and susceptibility to develop colorectal cancer has been investigated. Leucocyte DNA from 46 carcinoma patients and 49 controls was analysed by Southern blotting to determine the size distribution of restriction fragments containing the variable tandem repeat 3′ to the Ha-*ras* gene. Four predominant allelic fragments were found in both groups (in AvaII digests having sizes of 1.55, 2.0, 2.65 and 3.15 kilobases [kb]), together with a variety of 'rare' alleles (with individual frequencies <5%). The overall prevalence of rare alleles was not significantly different between cancer and control groups. The distribution of the common alleles, however, differed significantly. The combined frequency of the two larger alleles (a3 and a4) was approximately twice as high in the cancer group (34%) as in controls (18%) ($P<0.025$), which was reflected in a highly significant increase in the proportion of individuals carrying an a3 or a4 allele.

Activation of the *ras* family of oncogenes (Ha-, Ki- or N-*ras*) has been demonstrated in around 10–20% of randomly-selected human tumours (Balmain, 1985; Marshall, 1986). In most cases the mechanism of activation appears to be a somatic point mutation at one of a few critical sites (codons 12, 13 or 61) and two recent studies have revealed a particularly high incidence (up to 40%) of such a mutation in human colonic cancer DNA (Bos *et al.*, 1987; Forrester *et al.*, 1987). Oncogene activation can, however, result not from changes in the coding region but from inappropriate expression of an otherwise normal gene product, as in the case of c-*myc* in B cell lymphomas (Leder *et al.*, 1983). There is increasing evidence that such quantitative changes may also be involved in *ras* activation. Experimentally, elevation of expression can be sufficient to permit a normal Ha-*ras* gene to transform NIH3T3 cells (Chang *et al.*, 1982) and to immortalise primary fibroblasts (Spandidos & Wilkie, 1984). Direct observations on human material have revealed apparent increases in *ras* mRNA or p21 protein content in tumour *versus* normal cells in colon (Thor *et al.*, 1984; Spandidos & Kerr, 1984; Gallick *et al.*, 1985), as well as breast (Ohuchi *et al.*, 1986) and lung cancers (Kurzrock *et al.*, 1986). In contrast to c-*myc*, however, no structural changes in regulatory sequences have been identified in tumour *ras* genes which could account for an increase in expression as a somatic event.

In addition to *somatic* changes in oncogene structure, confined to tumour cells, however, the possibility must also be considered that *constitutional* differences in oncogene structure and/or expression could influence the likelihood of malignant transformation, by determining, for example, the number of additional somatic events required for tumourigenesis in any given tissue. Southern blot analysis of germline human Ha-*ras* genes (Goldfarb *et al.*, 1982) shows frequent restriction fragment length polymorphisms due to variation in the copy-number of a tandemly-repeated 28 bp sequence located ~1 kb downstream from the gene (Capon *et al.*, 1983). This variable tandem repeat (VTR) has been proposed as a possible candidate for a flanking sequence which might influence *ras* gene expression (Figure 1).

Krontiris *et al.* (1985) in a large survey of Ha-*ras* RFLPs in leucocyte (germ-line) DNA from patients with a variety of cancers together with unaffected controls found that whereas in both groups the majority (>85%) of allelic restriction fragments fell into just four 'common' sizes, in the cancer population there was a significantly higher prevalence (11.6% *vs.* 3.9%) of 'rare' fragment sizes, some of which

**Figure 1** Schematic representation of the human c-Ha-*ras* gene locus. Exons are represented by open boxes and the variable tandem repeat region by the hatched box. Recognition sites for restriction endonucleases are shown for BamH1 (B) and AvaII (A).

were never observed in the control group. It was suggested that the existence of these rare alleles might reflect an inherited abnormality, either specific to the Ha-*ras* locus or perhaps representing a more general genomic instability, which predisposed to the development of cancer.

Since the risk of malignancy may be cell-type specific the observed effect in this and a later series of mixed cancers (Krontiris *et al.*, 1986) may have been seriously 'diluted'. We and others (Thein *et al.*, 1986; Heighway *et al.*, 1986; Gerhard *et al.*, 1987) have therefore carried out a similar analysis of patients with a single tumour category. We chose to investigate colorectal cancer since it is one of the commonest in Western countries and has a particularly strong familial tendency (around a quarter of patients have a first-degree relative with the same disease – Lovett, 1976).

### Materials and methods

### *Subjects*

Forty-six patients who had undergone surgery for histologically-confirmed carcinoma of the colon or rectum were studied, together with 49 healthy controls with no family history of cancer in first-degree relatives.

### *Tumours*

Eighteen tumours were situated in the rectum, 14 in the sigmoid colon, 6 in the caecum, 3 in the descending and 3 in the ascending colon. The majority (40) were standard adenocarcinomas; 6 were classified as mucinous. Two were well-, 33 were moderately- and 11 were poorly-differentiated as defined by standard histopathological criteria (Dukes, 1949). Four were at Dukes' stage A, 20 at stage B and 19 at stage C (2 were examined by biopsy only and 1 was inoperable).

### *DNA extraction*

DNA was prepared from peripheral blood leucocytes by the method of Kunkel *et al.* (1977) modified by the addition of sodium perchlorate (to 1 M) after the proteinase K digestion step.

Correspondence: D. Wynford-Thomas.
Received 6 August 1987; and in revised form, 8 October 1987.

*Probe*

The plasmid used was pbcN-1 (Pulciani *et al.*, 1982) which contains a 6.6 kb human genomic BamH1 fragment including the c-Ha-*ras* 1 gene and 3′ VTR region.

*Southern blot analysis*

Genomic DNA (5–10 μg per lane) was digested with restriction endonuclease BamH1 or AvaII, fractionated on 0.6% or 1.0% agarose gels respectively and blotted to nylon membranes (Hybond, Amersham, UK). The 6.6 kb insert of pbc-N1 was $^{32}P$-labelled to a specific activity of $\sim 10^9$ cpm $\mu g^{-1}$ using the random primer method (Feinberg & Vogelstein, 1983). Hybridisation and washing were carried out as recommended by the manufacturers to a final stringency of 0.1 × SSC at 65°C. Membranes were autoradiographed at −70°C for 1–3 days. Fragment sizes were determined from the migration of co-electrophoresed HindIII restriction fragments of bacteriophage λ.

*Statistical analysis*

The significance of differences in allele frequency were assessed by the chi-square test, corrected for continuity where appropriate (Armitage, 1971).

**Results**

Initial analysis with BamH1 (Figure 2A) showed 4 common restriction fragment sizes of 6.6, 7.0, 7.6 and 8.0 kb, representing alleles designated a1, a2, a3 and a4. However, the large size of these fragments relative to the VTR seriously limited resolution so that alleles varying in size by <0.2 kb could not be reliably distinguished. Accordingly, the major study of allele size distributions was based on samples digested with AvaII, which cuts just outside the VTR, generating fragments for alleles a1 to a4 of 1.55, 2.0, 2.65 and 3.15 kb respectively. The resulting improvement in resolution is evident from comparison of Figures 2A and 2B. All samples were nevertheless also analysed by BamH1 digestion to ensure that variations in allele size observed in AvaII digests were indeed due to variations in the length of the VTR and not to a restriction site polymorphism for AvaII, which is occasionally found (Thein *et al.*, 1986).

Several sources of variability which could affect the precision of AvaII fragment size measurement were considered. Inter-gel variation was overcome by including samples representing the known common alleles (a1 to a4) in each gel to act as an internal standard (in addition to the HindIII markers). Within a given gel, significant variation in apparent fragment size was observed to result in early studies from variation in DNA loading; an apparent decrease of as much as 0.1 kb could result from a 3-fold increase in loading. Care was taken therefore to load equal $O.D._{260}$ units of sample on each lane and to further check for equality of loading by inspection of the ethidium bromide-stained gel prior to blotting.

With these precautions, comparison of samples from different patients having the same genotype run on the same gel gave a mean and standard deviation (s.d.) of fragment

**Figure 2** Comparison of the 4 'common' Ha-*ras* alleles in leucocyte DNA digested with BamH1 (A) or AvaII (B) and fractionated on 0.6% (A) or 1% (B) agarose gels respectively. The alleles shown are: lane a: a3, a2, a1 (composite of two samples used as internal standard); lane b: a4, a1; lane c: a1, a1; lane d: a3, a1. (M: size markers in kb.)

size for alleles a1, a2, a3 and a4 of 1.55+0.02 kb, 2.0+0.04 kb, 2.65+0.06 kb and 3.15+0.10 kb respectively. These correspond to 95% confidence intervals (2 × s.d.) for determination of these allele sizes, of 0.04 kb, 0.08 kb, 0.12 kb and 0.2 kb respectively. We were able, for example, to reproducibly distinguish between alleles in the 1.5–2.0 kb range which differed by only 100 bp (e.g., a5 *vs*. a6 in Figure 3). A 'rare' allele was defined as having a fragment size >2 s.d. from the nearest 'common' allele (a1, a2, a3 or a4).

On this basis five rare alleles with sizes from 1.6 kb to 2.8 kb were detected (Table I; Figure 3). The overall frequency of rare alleles was 2.0% (2/98) in the control population compared with 7.6% (7/92) in the cancer patients. This difference was not statistically significant ($\chi^2 = 2.1$; $P > 0.1$). However, comparison of the frequencies of all alleles between cancer and normal groups revealed a significant overall difference ($\chi^2 = 10.5$; $P < 0.05$). Further analysis showed that the two largest common alleles, a3 and a4, were much more frequent in cancer patients, the combined frequency of a3 plus a4 being almost twice that in controls (31/92 *vs*. 18/98; $\chi^2 = 5.05$; $P < 0.025$). This was reflected in a highly significant increase in the proportion of individuals carrying an a3 or a4 allele in the cancer group (29 out of 46) compared with the normals (15 out of 49) ($\chi^2 = 8.8$; $P < 0.01$). No other significant difference of either single or combined allele or genotype frequency was observed.

Sub-division of the cancer group by differentiation grade or clinical stage showed no correlation of rare allele frequency with any sub-type. The combined prevalence of the a3 and a4 alleles increased with decreasing differentiation but this failed to reach statistical significance (Table II).

**Figure 3** Common and rare Ha-*ras* alleles in control and cancer patients. Leucocyte DNA from control (lane a) and from colorectal cancer patients (lanes b to g) were digested with AvaII and fractionated on an 0.6% gel. The represented genotypes are – lane a: a4, a1; lane b: a8, a1; lane c: a4, a1; lane d: a4, a5; lane e: a4, a3, a1*; lane f: a3, a1; lane g: a3, a6. Rare alleles (a5, a6 and a8) are arrowed. (M: size markers in kb.)

*This is an internal standard composed of 2 samples (a4/a1 and a3/a1).

**Table I** Comparison of Ha-*ras* allele frequencies in patients with colorectal cancer and in an unaffected population

| | | | *Frequency*[a] | |
|---|---|---|---|---|
| | *Allele* | *Size (kb)* | *Cancer patients* | *Unaffected controls* |
| Common | a1 | 1.55 | 48 (53) | 68 (69) |
| | a2 | 2.0 | 6 (6.5) | 10 (10) |
| | a3 | 2.65 | 18 (20) | 11 (11) |
| | a4 | 3.15 | 13 (14) | 7 (7.1) |
| Rare | a5 | 1.6 | 1 (1.1) | – |
| | a6 | 1.7 | 4 (4.4) | 1 (1) |
| | a7 | 2.2 | 1 (1.1) | – |
| | a8 | 2.78 | 1 (1.1) | – |
| | a9 | 2.8 | – | 1 (1) |
| | | Total | 92 (100) | 98 (100) |

[a]Figures in brackets are percentages of the totals.

### Discussion

Our analysis of Ha-*ras* RFLPs in patients with a single tumour type – colorectal carcinoma – shows that in contrast to the results of a larger survey of mixed tumour categories (Krontiris *et al*., 1985) the frequency of rare alleles was not significantly increased in germline (leucocyte) DNA from the cancer group compared with a control population. Krontiris' studies showed a similar incidence in controls to our own, i.e., 3.9% (Krontiris *et al*., 1985) and 3.4% (Krontiris *et al*., 1986), but a highly significant increase ($P < 0.01$ and $P < 0.001$ respectively) in the cancer patients, which was not diminished when the solid tumour sub group was considered separately. (The majority of cases were leukaemias.) While our study was proceeding other similar analyses of specific tumours were reported. Thein *et al*. (1986) found rare alleles at a frequency of 2.8% in patients with myelodysplastic syndrome (MDS) compared with 4.8% in their controls. Gerhard *et al*. (1987) reported a rare allele prevalence of 4% in melanoma against 6% in controls, an interesting contrast to the 43% (6/14) seen in the Krontiris series. Similarly Heighway *et al*. (1986) reported figures of 5 to 7% in lung cancers compared with 4% in controls. All three therefore agree with our failure to demonstrate any significant over-representation of rare alleles in tumour patients. One striking exception is the report by Liderau *et al*. (1986) of an apparent increase in rare allele frequency from 9% in controls to 41% in breast cancer patients.

Although more surveys will be needed before any final conclusion can be reached, it is already clear therefore that the association of rare Ha-*ras* alleles with cancer predisposition does not hold for most tumour types so far examined. It should be pointed out, however, that the importance of considering all sources of artefact affecting the migration and hence apparent size of fragments in these Southern blot analyses has not been sufficiently stressed. We observed in preliminary studies, for example, that leucocyte and tumour DNA samples obtained from the same patients,

**Table II** Combined frequencies of a3 plus a4 alleles in unaffected controls and in patients with colorectal cancer of different grade and stage

| | | *Frequency*[a] |
|---|---|---|
| | Unaffected controls | 18/98 (18) |
| | Cancer patients: | |
| Grade | Poorly differentiated | 9/22 (41) |
| | Moderately differentiated | 21/66 (32) |
| | Well differentiated | 1/4 (25) |
| Stage | Dukes' A | 5/8 (63) |
| | Dukes' B | 14/40 (35) |
| | Dukes' C | 12/38 (32) |
| | All cancer patients | 31/92 (34) |

[a]Frequency of a3 plus a4 as proportion of total alleles in each group; Figures in brackets are percentages.

but extracted (for other reasons) by different methods, frequently showed a slight difference in migration corresponding to an apparent size difference of up to a few hundred base pairs. This could be particularly important where comparison is based on leucocyte DNA for controls but tumour DNA for cancer patients (e.g., Liderau *et al.*, 1986). The finding of an apparent rare/rare genotype, in which a similar deviation from normal is observed in both alleles, could well be due to such an artefact.

Although no change in the incidence of rare alleles was seen in our study, a significant change in the relative abundance of the four normal alleles was observed, consisting of an increase in the rarer a3 and a4 alleles with a corresponding fall in the frequency of a1 and a2. In contrast to the increased a3 plus a4 frequency from 18 to 34% seen here in colorectal cancer patients, no increases were reported in the mixed tumour series (or solid tumour subgroup) of Krontiris *et al.* (1985) or in the MDS, melanoma and breast studies referred to above. Our result agrees closely however with the observation of a similar magnitude increase (15–29%) in the incidence of the a4 allele in one sub-type of lung cancer – non-small cell carcinoma (Heighway *et al.*, 1986).

There are at least two possible explanations for this finding. First it may represent an *indirect* association in which the longer, a3 or a4, alleles are in linkage disequilibrium with a cancer-predisposing allele of a neighbouring locus. Alternatively, it may represent a true *direct* association in which the increased length of VTR in some way contributes to an increased risk of developing cancer. It has been suggested that the VTR region may have an enhancer-like activity since its removal leads to a decrease in transforming efficiency of mutant Ha-*ras* gene clones (Krontiris *et al.*, 1985) and to a decreased expression of the normal gene (Seeburg *et al.*, 1984). It is possible therefore that gene expression is increased by a lengthening of the VTR. It is unlikely, however, that any of the RFLPs at the Ha-*ras* locus play a direct role in generating the tumour phenotype; none of the common or rare Ha-*ras* alleles shows transforming activity in the NIH3T3 assay for example (Krontiris *et al.*, 1985). More likely, possession of a given allele modulates the action of the primary oncogene abnormalities (for example Ki-*ras* mutation) thereby acting as one of a multiple set of hereditary factors which combine to influence the risk of tumour development.

We are grateful to the Cancer Research Campaign for grant support, and to Mr B.I. Rees and Mr H.L. Young for access to clinical samples.

### References

ARMITAGE, P. (1971). *Statistical Methods in Medical Research.* Blackwell: Oxford.

BALMAIN, A. (1985). Transforming ras oncogenes and multistage carcinogenesis. *Br. J. Cancer*, **51**, 1.

BOS, J.L., FEARON, E.R., HAMILTON, S.R. & 4 others (1987). Prevalence of ras gene mutations in human colorectal cancers. *Nature*, **327**, 293.

CAPON, D.J., CHEN, E.Y., LEVINSON, A.D., SEEBURG, P.H. & GOEDDEL, D.V. (1983). Complete nucleotide sequence of the T24 human bladder carcinoma oncogene and its normal homologue. *Nature*, **302**, 33.

CHANG, E.H., FURTH, M.E., SCOLNICK, E.M. & LOWY, D.R. (1982). Tumorigenic transformation of mammalian cells induced by a normal human gene homologous to the oncogene of Harvey murine sarcoma virus. *Nature*, **297**, 479.

DUKES, C.E. (1949). The surgical pathology of rectal cancer. *J. Clin. Pathol.*, **2**, 95.

FEINBERG, A.P. & VOGELSTIEN, B. (1983). A technique for radiolabelling DNA restriction endonuclease fragments to high specific activity. *Anal. Biochem.*, **132**, 6.

FORRESTER, K., ALMOGUERA, C., HAN, K., GRIZZLE, W.E. & PERUCHO, M. (1987). Detection of high incidence of K-ras oncogenes during human colon tumorigenesis. *Nature*, **327**, 298.

GALLICK, G.E., KURZROCK, R., KLOETZER, W.S., ARLINGHAM, R.B. & GUTTERMAN, J.V. (1985). Expression of p21ras in fresh primary and metastatic human colorectal tumours. *Proc. Natl Acad. Sci. USA*, **82**, 1795.

GERHARD, D.S., DRACOPOLI, N.C., BALE, S.J. & 5 others (1987). Evidence against Ha-ras-1 involvement in sporadic and familial melanoma. *Nature*, **325**, 73.

GOLDFARB, M., SHIMIZU, K., PERUCHO, M. & WIGLER, M. (1982). Isolation and preliminary characterisation of a human transforming gene from T24 bladder carcinoma cells. *Nature*, **296**, 404.

HEIGHWAY, J., THATCHER, N., CERNY, T. & HASLETON, P.S. (1986). Genetic predisposition to human lung cancer. *Br. J. Cancer*, **53**, 453.

KRONTIRIS, T.G., DI MARTINO, N.A., COLB, M. & PARKINSON, D.R. (1985). Unique allelic restriction fragments of the human Ha-*ras* locus in leucocyte and tumour DNAs of cancer patients. *Nature*, **313**, 369.

KRONTIRIS, T.G., DI MARTINO, N.A., COLB, M., MICHESON, D. & PARKINSON, D.R. (1986). Human restriction fragment length polymorphisms and cancer risk assessment. *J. Cell Biochem.*, **30**, 319.

KUNKEL, L.M., SMITH, K.D., BOYER, S.H. & 6 others (1977). Analysis of human Y-chromosome-specific reiterated DNA in chromosome variants. *Proc. Natl Acad. Sci. USA.*, **74**, 1245.

KURZROCK, R., GALLICK, G.E. & GUTTERMAN, J.U. (1986). Differential expression of p21 ras gene products among histological subtypes of fresh primary human lung tumours. *Cancer Res.*, **46**, 1530.

LEDER, P., BATTEY, J., LENOIR, G. & 5 others (1983). Translocations among antibody genes in human cancer. *Science*, **222**, 765.

LIDERAU, R., ESCOT, C., THEILLET, C. & 4 others (1986). High frequency of rare alleles of the human c-Ha-*ras*-1 proto-oncogene in breast cancer patients. *J. Natl Cancer Inst.*, **77**, 697.

LOVETT, E. (1976). Family studies in cancer of the colon and rectum. *Br. J. Surg.*, **63**, 13.

MARSHALL, C.J. (1986). The ras gene family. In *Oncogenes and Growth Control*, Kahn, P. & Graf, T. (eds) p. 192. Springer-Verlag: Berlin.

OHUCHI, N., THOR, A., PAGE, D.L., HORAN HAND, P., HALTER, S.A. & SCHLOM, J. (1986). Expression of the 21,000 molecular weight ras protein in a spectrum of benign and malignant human mammary tissues. *Cancer Res.*, **46**, 2511.

PULCIANI, S., SANTOS, E., LAUVER, A.V., LONG, L.K. & BARBACID, M. (1982). Transforming genes in human tumours. *J. Cell Biochem.*, **20**, 51.

SEEBURG, P.H., COLBY, W.W., CAPON, D.J., GOEDDEL, D.V. & LEVINSON, A.D. (1984). Biological properties of human c-Ha-ras-1 genes mutated at codon 12. *Nature*, **312**, 71.

SPANDIDOS, D.A. & KERR, I.B. (1984). Elevated expression of the human ras oncogene family in premalignant and malignant tumours of the colorectum. *Br. J. Cancer*, **49**, 681.

SPANDIDOS, D.A. & WILKIE, N.M. (1984). Malignant transformation of early passage rodent cells by a single mutated human oncogene. *Nature*, **310**, 469.

THEIN, S.L., OSCIER, D.G., FLINT, J. & WAINSCOAT, J.S. (1986). Ha-*ras* hypervariable alleles in myelodysplasia. *Nature*, **321**, 84.

THOR, A., HORAN HAND, P., WUNDERLICH, D., CARUSO, A., MURARO, R. & SCHLOM, J. (1984). Monoclonal antibodies define differential ras gene expression in malignant and benign colonic diseases. *Nature*, **311**, 562.